# PD-1 inhibitor in the treatment of relapsed primary mediastinal large B-cell lymphoma follow up by ^18^F-FDG PET/CT: A case report and literature review

**DOI:** 10.1016/j.radcr.2024.07.053

**Published:** 2024-08-02

**Authors:** ChiIeng Tou, LinFeng Ma

**Affiliations:** Department of Medical Imaging Center, Kiang Wu hospital, Macau, China

**Keywords:** Primary mediastinal large B-cell lymphoma, Relapsed, PD-1 inhibitor, Complete metabolic remission, ^18^F-FDG PET/CT

## Abstract

Primary mediastinal large B-cell lymphoma (PMBCL) is a specific subtype of diffuse large B-cell lymphoma (DLBCL), which occurs more frequently in young women. PMBCL is an uncommon kind of cancer. R-EPOCH is a common therapeutic regimen that is suitable for patients with PMBCL, and could get a relatively high complete remission rate. However, it may not be effective response in patients with relapsed PMBCL. Immunotherapy appears to be helpful in recent years. Therefore, in this case, a 31-year-old female patient with relapsed PMBCL. Progressive disease was identified after rechemotherapy and target therapy, complete remission can be achieved after switching to PD-1 inhibitor plus targeted therapy. These recurrence, progression, remission and follow-up are all displayed well on ^18^F-FDG PET/CT. This case with consecutive imaging monitor illustrates that PD-1 inhibitor may be used as a first-line treatment for recurrent PMBCL. In addition, ^18^F-FDG PET/CT is strongly recommended for monitoring PMBCL include baseline staging, interim response and follow-up study.

## Introduction

Primary mediastinal large B-cell lymphoma (PMBCL) is a rare subtype of lymphoma, clinically and biologically distinct from diffuse large B-cell lymphoma (DLBCL), that shows overlapping features with classical Hodgkin lymphoma (cHL) [1]. It is more common in young women [2]. The first-line treatment approaches for PMBCL result in a curability rate of 80–85%. Although recurrence might occur early in cases with chemorefractory disease, leading to a poor prognosis. The 9p24.1 rearrangement which causes increased expression of the immune checkpoint molecules PDL1 and PDL2, has facilitated the development of immune checkpoint blockers for various conditions. PD-1 inhibitors are authorized for treating relapsed/refractory cHL [3]. Here, we present a case of a 31-year-old female patient with relapsed PMBCL that is resistance to chemotherapy. Following treatment with a PD-1 inhibitor,^18^F-FDG PET/CT showed three times that there were no active lesions to suggest relapsed lymphoma. (DV score 1), indicating that relapsed/refractory PMBCL was effectively treated. Here is the case report.

## Case study

**Fig. 1:**
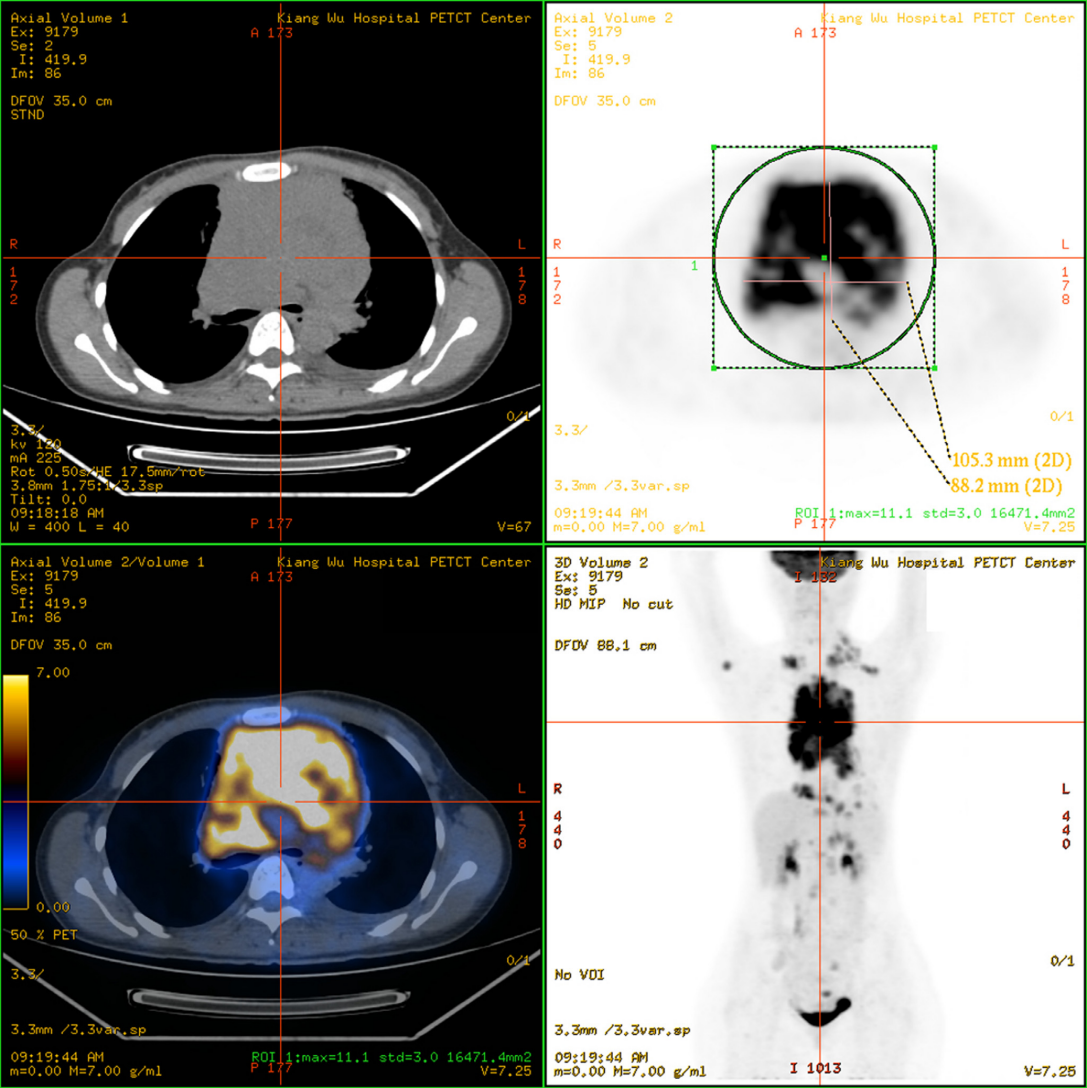
A 31-year-old female suffered from PMBCL with a baseline ^18^F-FDG PET/CT scan. An intensely hypermetabolic mass in the mediastinum accompanying multiple active lymph nodes, active lung lesions and bony foci.

**Fig. 2:**
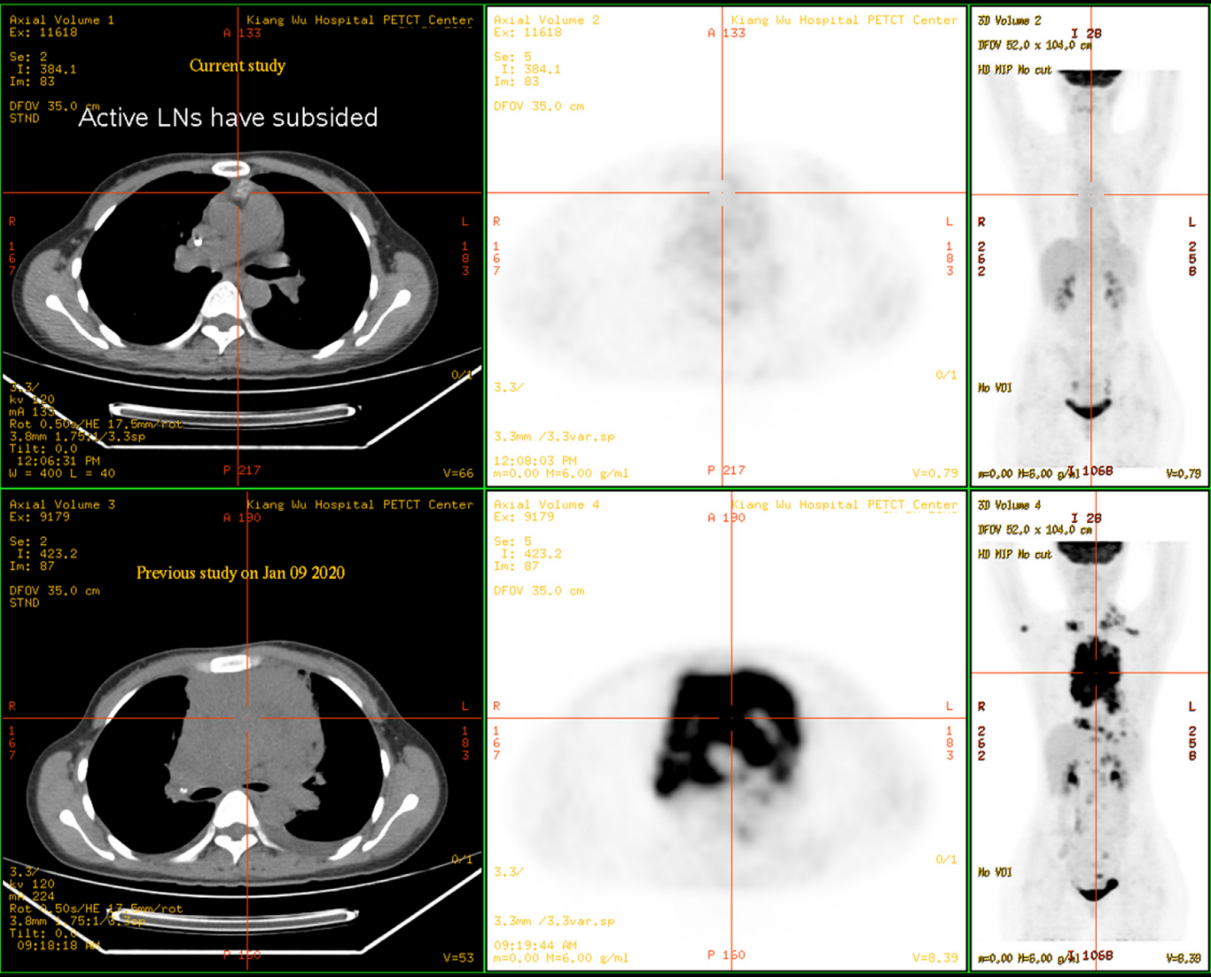
The ^18^F-FDG PET/CT scan showed complete metabolic remission. The following ^18^F-FDG PET/CT scan on September 24, 2020 indicated almost complete remission of the disease after 6 cycles R-EPOCH, 3 cycles HDMTX and 20 fractions of radiation therapy to the lung lesion.

**Fig. 3:**
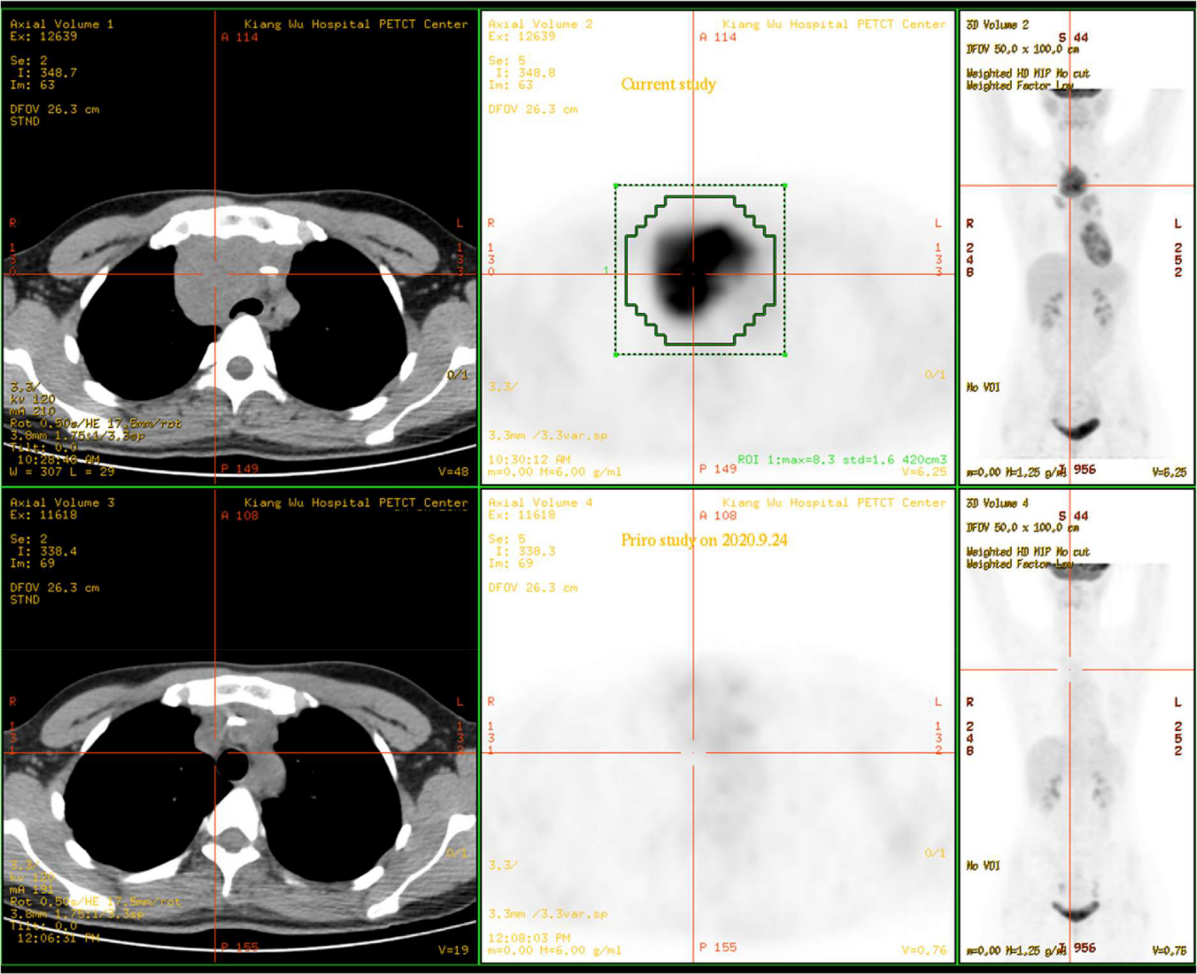
The ^18^F-FDG PET/CT scan showed relapsed disease in the mediastinal region. The ^18^F-FDG PET/CT scan on April 07, 2021 showed recurrent tumor with hypermetabolism in the mediastinum after half a year.

**Fig. 4:**
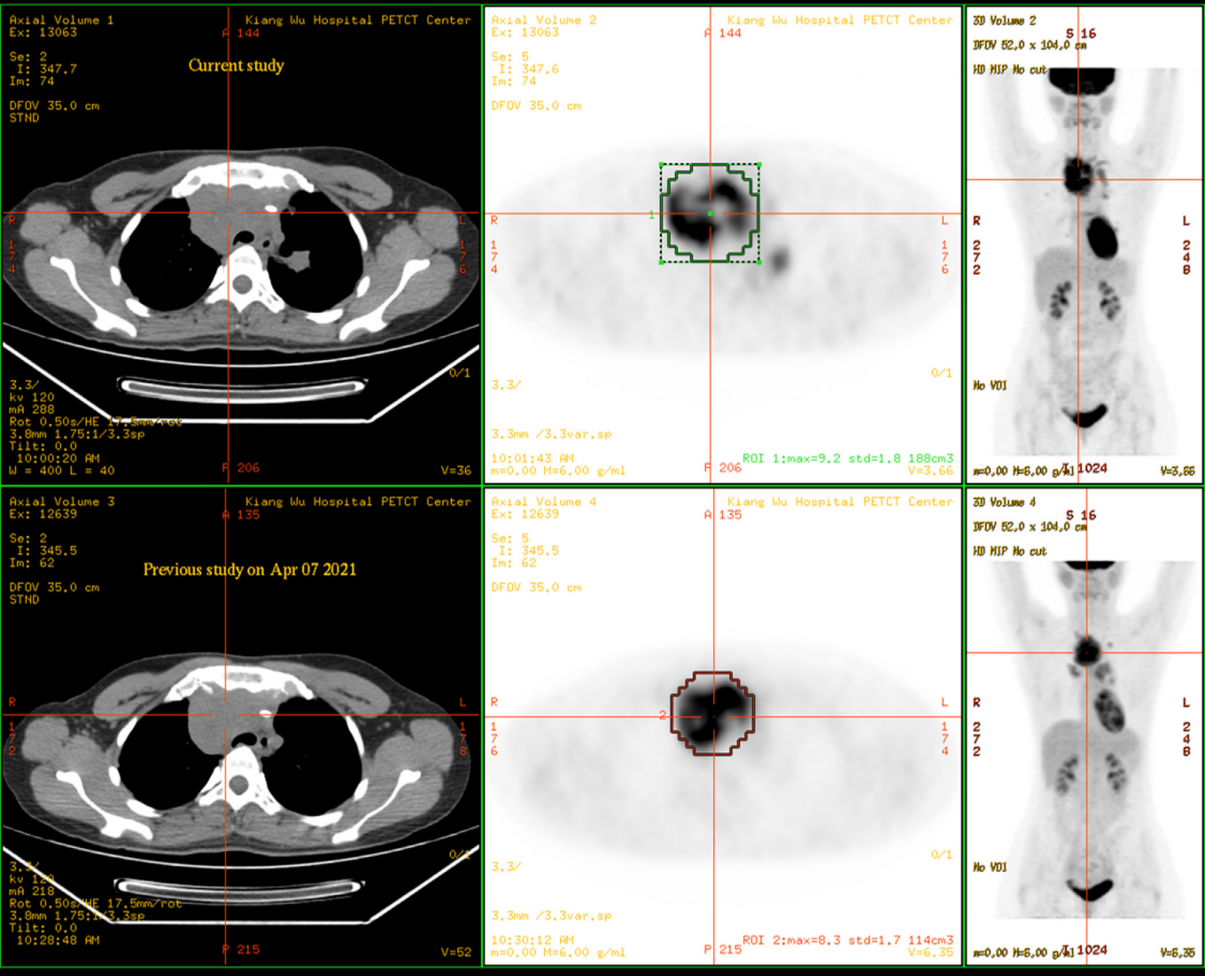
Progressive disease was identified after re-chemotherapy and target therapy. The work-up ^18^F-FDG PET/CT on June 26, 2021 showed worsening of the recurrent tumor with hypermetabolism in the mediastinum after re-chemotherapy and target therapy.

**Fig. 5:**
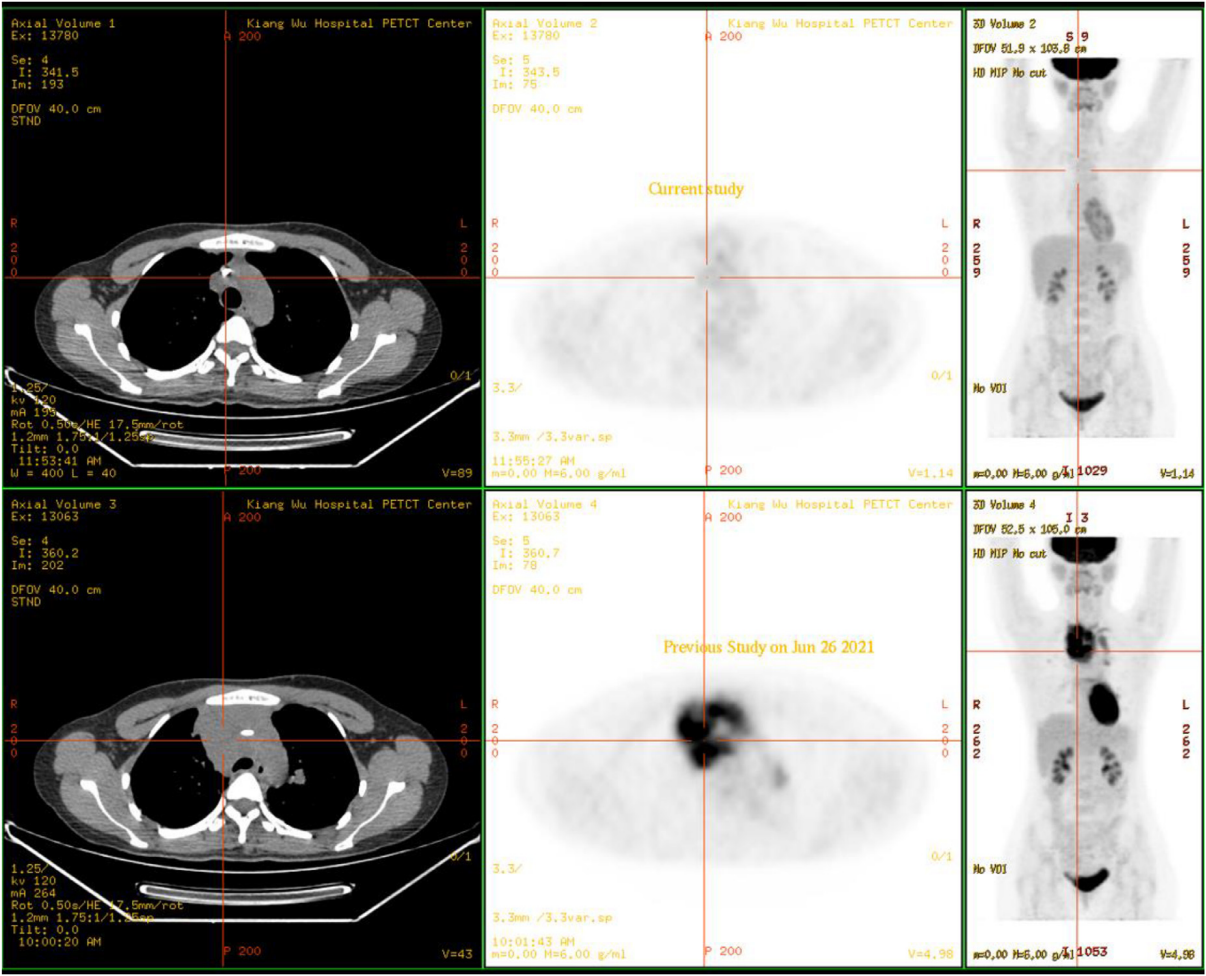
The ^18^F-FDG PET/CT scan showed complete metabolic remission. Post PD-1 inhibitor and targeted therapy, the ^18^F-FDG PET/CT scan on October 19, 2021 showed complete metabolic remission of the disease.

**Fig. 6:**
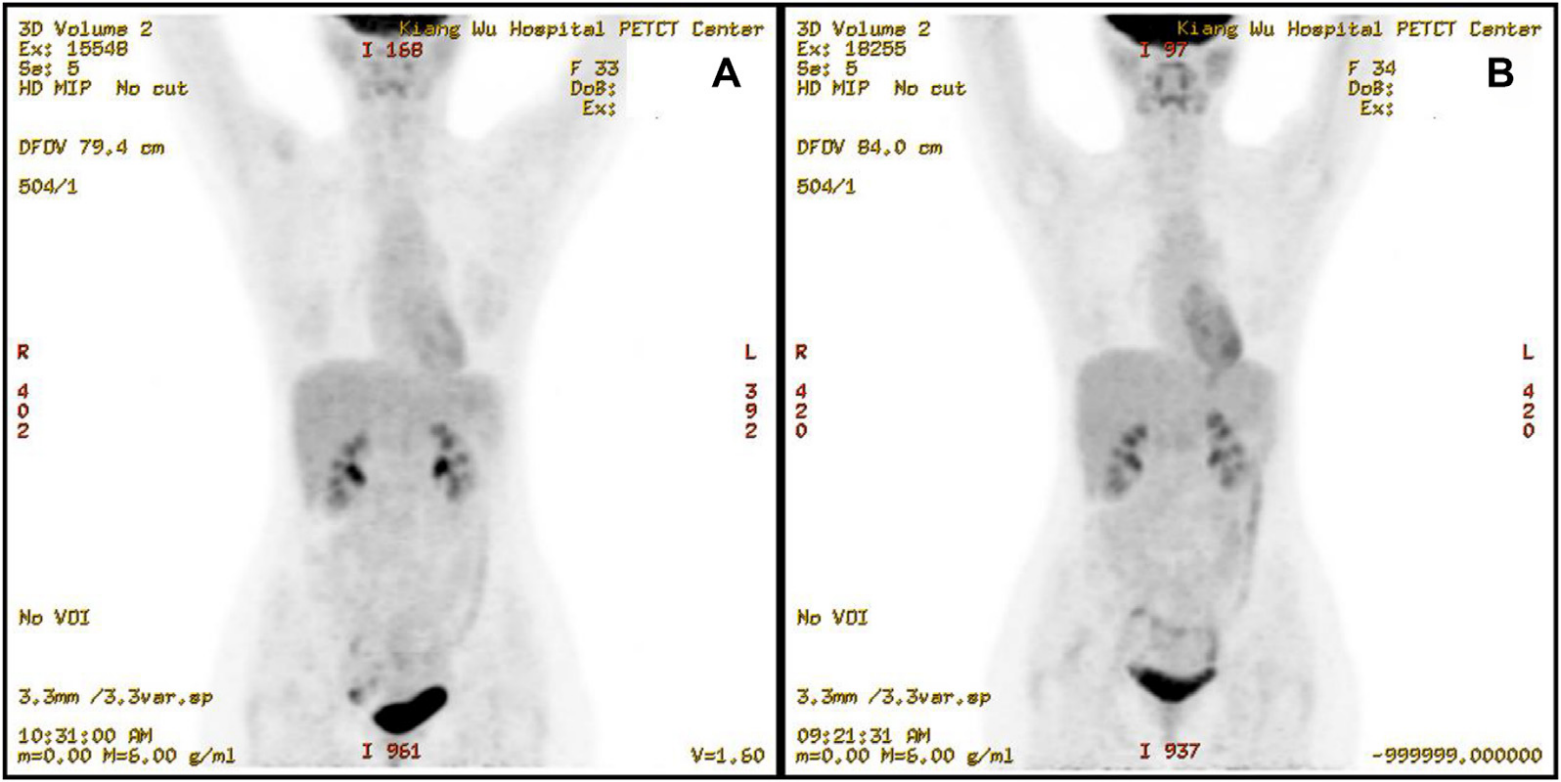
The ^18^F-FDG PET/CT scans showed complete metabolic remission twice again. (A) The ^18^F-FDG PET/CT scan on September 8, 2022 showed complete metabolic remission of the disease. (B) The ^18^F-FDG PET/CT scan on September 11, 2023 showed complete metabolic remission of the disease.

A 31-year-old female was admitted to Kiang Wu Hospital, with complaint of cough for 1 month. On the same day, she had neck ultrasound showed that multiple enlarged lymph nodes in bilateral neck and supraclavicular regions. The chest CT on hospital day 2 showed that the mass in the anterior and middle mediastinum accompanying with multiple enlarged lymph nodes. The tumor markers were checked: CA125 (461.00 U/mL, reference range <35 U/mL) was elevated, while the rest of AFP, CEA, CA15.3, CA19.9, and CA72.4 were normal. The baseline ^18^F-FDG PET/CT (Fig. 1) on hospital day 6 showed that a huge irregular hypermetabolic tumor in the anterior middle and upper mediastinum with localized necrosis. Multiple hypermetabolic enlarged lymph nodes were found on the both sides of diaphragm. 3. Bone metastases were identified in the right humeral head, thoracic 12 vertebrae, and the right ilium. Biopsy for the mediastinal mass confirmed PMBCL and clinical stage IVB. She underwent 6 cycles of R-EPOCH and 3 cycles of HDMTX at Hong Kong Sanatorium & Hospital. Followed by 20 fractions of radiation therapy to the lung lesion, the treatment was completed. The following ^18^F-FDG PET/CT scan (Fig. 2) indicated almost complete remission of the disease. After 6 months, there was a relapsed disease in the mediastinal region (Fig. 3). Biopsy again showed CD20+++ and CD30 100%, ki67 70%, PDL1-, PDL1(22C3)(TPS 70%+), EBERs-ISH-. Because of progressive disease was identified after rechemotherapy and target therapy (Fig. 4), she was switching to PD-1 inhibitor (Sintilimab 200MG D1) and targeted therapy (Brentuxumab 100MG D1) along with CTX 1.6g D1-3. After 2 weeks, CTX was suspended. The following ^18^F-FDG PET/CT scan (Fig. 5) indicated complete metabolic remission of the disease. Due to the COVID-19 epidemic, the patient was unable to go to China. Instead, she had targeted therapy (CD30 100MG) and received a PD-1 inhibitor (Keytruda 100MG) in Kiang Wu Hospital and Centro Hospitalar Conde de São Januário (CHCSJ) in Macau. Twice ^18^F-FDG PET/CT scans (1 year/2 years after treatment) (Fig. 6) conducted indicated metabolic complete remission.

## Discussion

PMBCL is an uncommon, fast-growing B-cell non-Hodgkin lymphoma that originates in the thymus and has unique clinical, pathological and genetic characteristics [4]. It typically manifests in young females and is clinically more prevalent in phases I to II [5]. PMBCL is characterized by a mass in the anterior mediastinal cavity that quickly spreads, causing symptoms such as pleural effusion, cough, difficulty breathing, and difficulty swallowing [6]. Extra-nodal organs may be involved at onset, including the liver, kidneys, gastrointestinal tract, and ovaries. The bone marrow involvement is uncommon [7]. CT, MRI, and ^18^F-FDG PET/CT are the main imaging techniques used to diagnose PMBCL. Typical imaging of the mass usually displays hypodensity characteristics such as hemorrhage, necrosis, or cystic degeneration at different levels. Additional imaging findings include of unilateral elevation of the diaphragm, pleural effusion, and pericardial effusion. Among them, ^18^F-FDG PET/CT is useful for examining the location, shape, density, necrosis, and calcification of lesions, as well as evaluating the degree of tumor involvement and residual tumor post-treatment [8]. However, it should be noted that ^18^F-FDG PET/CT has good negative predictive value but limited positive predictive value. Mediastinal inflammatory tissue and tissue reactivity after therapy can result in a significant number of incorrect positive results [9]. Therefore, PMBCL diagnosis is based on clinical characteristics and distinct pathological features. In this case, the patient was a 31-year-old female who presented clinically with an obvious mediastinal mass with multiorgan including many lymph nodes throughout the body, lungs, bones, uterus, and ovaries involvement, and was pathologically diagnosed with DLBCL (stage IVB). Studies have found that ^18^F-FDG PET/CT is useful in detecting recurrent lesions. In this case, disease recurrence in the mediastinal region was identified by ^18^F-FDG PET/CT after chemotherapy and radiotherapy. Some studies noted that while most newly diagnosed patients can be successfully treated with multiagent chemoimmunotherapy, the prognosis for patients with relapsed/refractory PMBCL is poor, especially for those who cannot undergo or experience a relapse after second-line autologous stem-cell transplantation due to the aggressive and chemotherapy-resistant nature of the disease. Disease recurrence typically happens in the early stage, with relapse-refractory patients making up 10% to 30% of all patients [10]. PMBCL is believed to be vulnerable to PD-1 blockage due to genetic abnormalities at 9p24 and increased expression of PD-L1 [11]. Blocking the route can be used to treat the condition. Therefore, patient's treatment regimen was switched to PD-1 inhibitor. She received treatment with Sintilimab (domestic) and Keytruda both of which are PD-1 inhibitors used in the treatment of Hodgkin's lymphoma, which can be combined with programmed death protein 1 (PD-1) and have been approved by the FDA for use in patients with PMBCL who have progressed after 2 or more treatment regimens, which has a high response rate, long-lasting activity, and a manageable safety profile [12]. Combining PD-1 monoclonal antibody with chemotherapy can enhance effectiveness. Furthermore, the ^18^F-FDG PET Deauville score can be used to identify patients who can be treated without subsequent radiotherapy [13]. Deauville-negative (scores of 1-3) patients are closely followed up for observation and are not intervened before disease progression. Following this patient's disease relapse, the treatment plan was altered, and three successive ^18^F-FDG PET/CT scans indicated that no active lesion to suggest lymphoma was identified. (Deauville score 1) and the pulmonary nodules and opacities were stable in size and metabolic quiescence (Deauville score 1). It means PMBCL show metabolic complete remission.

Due to the lack of sufficient prospective randomized clinical trials, it is difficult to derive a uniform and effective protocol for the treatment of PMBCL. The outlook for relapsed/refractory PMBCL is unfavorable. While most patients are successfully treated with standard chemotherapy initially, the chances of morbidity and mortality increase significantly if PMBCL recurs. Hence, it is essential to promptly modify the treatment strategy and create alternative strategies through follow-up visits. The study discovered lesion recurrence in the patient using ^18^F-FDG PET/CT. The patient experienced improved results after getting a PD-1 inhibitor based on ^18^F-PET/CT follow-up, suggesting a PD-1 inhibitor may be used as a first-line therapeutic approach for relapsed PMBCL patients. In addition, ^18^F-FDG PET/CT is strongly recommended for monitoring PMBCL include baseline staging, interim response, and follow-up study.

## Ethical approval

All procedures performed in studies involving human participants were in accordance with the ethical standards of the institutional research committee and with the 1964 Helsinki declaration and its later amendments or comparable ethical standards.

## Patient consent

Written informed consent for the publication of this case report was obtained from the patient.
